# Proposition of Hyper‐Chemical Exchange Saturation Transfer Subtraction Spectroscopy to Detect Very Weak and Broad Signals Hidden Under Baseline and Widen Range of Materials Accessed by Hyperpolarized ^129^Xe NMR

**DOI:** 10.1002/cphc.202500249

**Published:** 2025-10-26

**Authors:** Hideaki Fujiwara, Hirohiko Imai, Atsuomi Kimura

**Affiliations:** ^1^ Department of Medical Physics and Engineering Division of Health Sciences, Graduate School of Medicine Osaka University 1‐7 Yamadaoka Suita Osaka 565‐0871 Japan; ^2^ Innovation Research Center for Quantum Medicine Gifu University School of Medicine 1‐1 Yanagido Gifu 501‐1194 Japan

**Keywords:** free volumes, hidden signals, NMR spectroscopies, polyimide and gelatin, xenon

## Abstract

A recirculating hyperpolarization system is used for the hyper‐Chemical Exchange Saturation Transfer (CEST) experiment after a stopped‐flow scheme and a subtraction mode are added. The resulting hyper‐CEST subtraction spectroscopy (HCSS) is effective in providing evidence for weak or broad signals. The chemical shift of these broad and weak signals has been successfully determined thanks to a thorough examination of the saturation frequency dependency. An application to polyimide reveals a very broad signal at 206.5 ppm with a line width of 96 ppm (11 kHz). The tiny dimension of the void space in polyimide, where the embedded Xe atoms are in a severely constrained state in dynamics, is supported by this broadness combined with the low field chemical shift. The weak signal that is not easy to be made evident in the standard hyperpolarized ^129^Xe spectrum is successfully observed with additional application to gelatin capsules. As the storage year progresses on, the chemical shift determined for this sample reveals a low field shift.

## Graphical abstract

A polyimide sample, for which hyperpolarized 129Xe NMR spectroscopy failed to detect 129Xe embedded in the polymer (Inset A), is found to give a very broad peak, line width 11 kHz at 206.5 ppm, by applying the stopped‐flow hyper‐chemical exchange saturation transfer subtraction spectroscopy on the continuous‐flow recirculating hyper‐polarization system (Inset B).

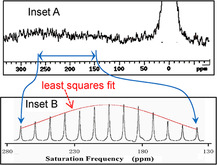

## 1 Introduction

NMR spectroscopy is an effective method for studying the dynamics, structure, and function of polymers. Important information about the voids space in polymers, also known as free volume, can be obtained via ^129^Xe NMR. This information is directly linked to physical characteristics like electrical and transport properties, viscosity, and heat reactivity.^[^ ^1^, ^2^ ^]^ Numerous techniques, such as electrochromic spin probes, small‐angle X‐ray and neutron diffractions, positron annihilation lifetime spectroscopy (PALS), and ^129^Xe NMR spectroscopy, have been used to investigate the free volume in polymers.^[^ ^3^ ^]^ Recent advancements in the 2D technique have demonstrated that ^129^Xe NMR has notable characteristics as an advanced technology that may reveal dynamic profiles of ^129^Xe spins trapped in various medium environments. This technique is gaining growing attention in the analysis of composite materials.^[^ ^4^, ^5^ ^]^ The introduction of the hyperpolarization technique is another that may be more significant in the recent evolution of NMR spectroscopy.^[^ ^6^ ^]^ For nuclei like ^13^C, ^15^N, and ^129^Xe, this approach implements an exceptional improvement in NMR sensitivity. In comparison to its thermal equilibrium condition, the enhancement factor for ^129^Xe can reach up to five orders of magnitude. A highly advanced method known as hyper‐chemical exchange saturation transfer (CEST)^[^ ^7^ ^]^ has been developed to significantly improve the performance of hyperpolarized (HP) ^129^Xe NMR and magnetic resonance imaging by incorporating the CEST effect. According to reports, hyper‐CEST has so far proved effective for the solute such as Xe host molecules in identifying concentrations that are subnano‐ or picomolar.^[^ ^8^, ^9^, ^10^ ^]^ A thorough method of analysis for the saturation frequency dependence in the hyper‐CEST subtraction spectroscopy (HCSS) was recently proposed by the authors on the continuous‐flow/recirculating hyperpolarization system, which has made possible the chemical shift determination of very weak signals that are not easy to detect by the standard HP ^129^Xe NMR.^[^ ^11^ ^]^ The subtraction mode in HCSS can suppress the symmetric part of the bulk signal in the case of partial overlap with other signals of interest as well as provide a possibility to reduce potential HP fluctuation and drift effects. It also changes phase of the signals in the spectra from negative to positive, likewise in the ordinary NMR spectra.

The goal of this study is to demonstrate the potential usefulness of the developed HCSS in releasing the limitation of materials accessed by ^129^Xe NMR and in assessing the void space in polymers as a crucial indicator for the physical characteristics of materials. In the HCSS method, here, solute signal of ^129^Xe embedded in polymers is saturated, and the saturation is transferred to bulk gas signal. The gas signal possesses a long relaxation time and is suited for accumulating numbers of saturations transferred, leading to an effective enhancement of sensitivity. By sweeping the saturation frequency within a range of interests by accumulating an appropriate number of fids, new peak will appear if the chemical shift is included in the range. Here, the bulk gas peak which gives a reference to the chemical shift needs not be included in the range since it can be obtained simply by a separate measurement.

Subtraction of the hyper‐CEST response can help promoting the stability of HCSS. Especially in the hyperpolarized nucleus, relaxation and depolarization disturb the stability of output signals, and hence subtraction in a short period of time will enhance the stability or reproducibility of the experiment. This is important when a detailed method of analysis is applied to the experimental data. When a reference is set to the mirror point of the bulk gas signal, interferences from the gas peak can be suppressed at least for the symmetric part of the gas peak even if the range of interests is not far from the gas peak.

Polyimide and gelatin are the target molecules selected as model compounds for this study. Moreover, due to its high thermotolerance—which includes high mechanical strength, insulating qualities, low dielectric constant and dielectric loss, and resistance to chemicals and radiation at high temperatures—polyimide is a material that is appealing to the industry.^[^ ^12^, ^13^ ^]^

The pharmaceutical industry uses gelatin extensively because it is a versatile ingredient. For example, it is a valuable ingredient used to make the soft and hard gelatin capsules.^[^ ^14^ ^]^ Additionally, it is commonly used as a convenient ingredient in a variety of foods.

## 2 Results and Discussion

**Figure 1:**
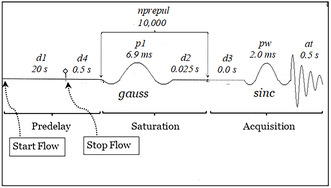
The stopped‐flow hyper‐CEST pulse sequence used in the present study. Shaped pulses, gauss and sinc, are used. *d1*−*d4* mean delay times. at is acquisition time. *p1* and pw denote pulse width, and nprepul is the number of saturation pulses. Subtraction is made every even number of fid acquisition while the saturation frequency alternates between reference and measurement frequencies.

**Figure 2:**
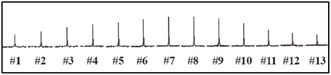
The saturation frequency dependence in HCSS of polyimide. The saturation frequency was changed from 266.5 to 147.2 ppm in the Increment of 9.904 ppm (1100 Hz) from left (#1) to right (#13).

**Figure 3:**
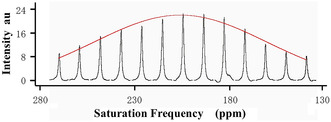
Saturation frequency dependence of polyimide measured in HCSS as an envelope of the spectra. The red curve shows a least squares fitting whose top corresponds to the chemical shift.

Although the standard hyperpolarized ^129^Xe spectrum (Figure S1, Supporting Information) was unable to identify any signals assignable to ^129^Xe entrapped in polyimide tube, Flon Industry Inc., Japan, application of HCSS by use of the pulse sequence developed in the laboratory (**Figure** **1**) revealed a very wide distribution of the saturation frequency as shown in **Figure** **2**, where saturation frequency was changed stepwise from #1 to #13 spectrum. Each spectrum represents the peak height at each saturation frequency in the very broad peak of the entrapped ^129^Xe. Therefore, the broad peak can be reproduced from a least squares fitting of the saturation frequency dependence (**Figure** **3**). Here, the chemical shift, δ, is obtained from the saturation frequency value corresponding to the top of the resulting fitted curve, f_top_, after subtracting the saturation frequency for the bulk gas, *f*_bulk_, i.e., *δ* = (*f*_top _− *f*_bulk_)/*ν* where *ν* represents the Larmor frequency set on the spectrometer, 110.624 MHz. The *f*_bulk_ value can be simply obtained as the saturation frequency for the bulk gas peak by observing the gas peak just before or after the experiment of HCSS.

**Figure 4:**
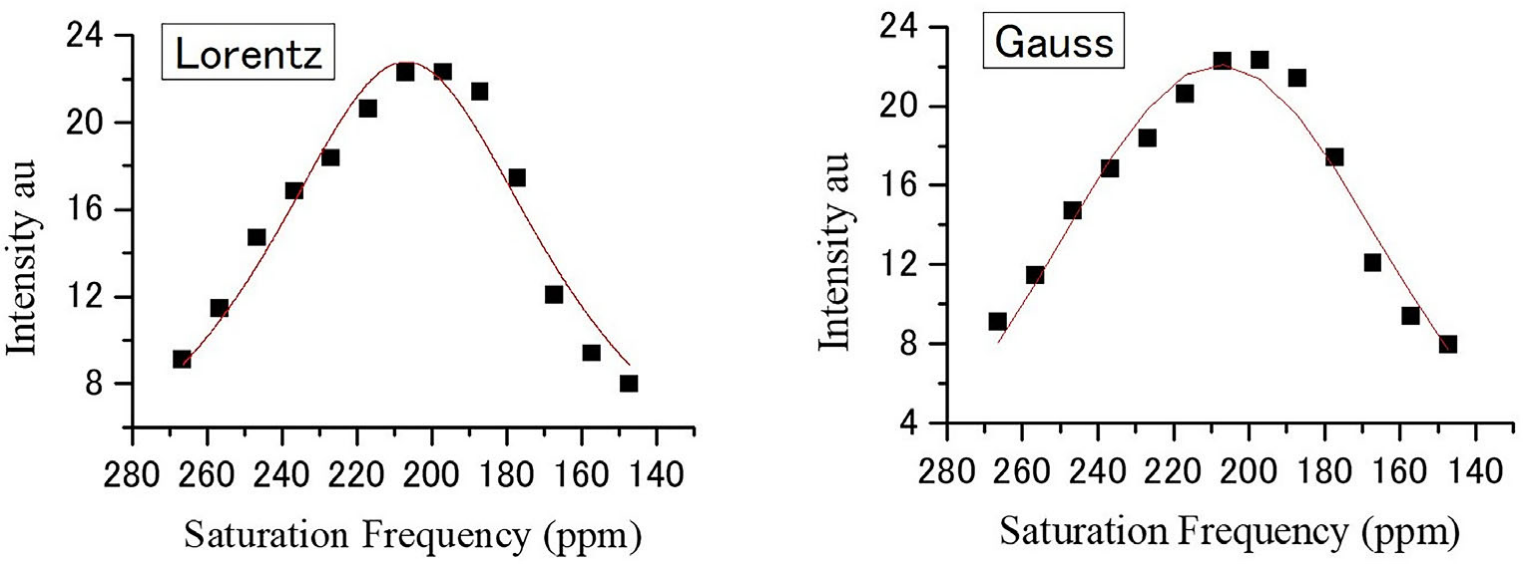
Analysis of the saturation frequency dependence of polyimide measured in HCSS. The curve denotes least squares fit.

The analysis of this frequency dependence was made on the least squares scheme by assuming Lorentz or Gauss function, Equation (1) or Equation (2), respectively, for the total line shape.

(1) $$y=y_{0}+\;\left(\frac{2A}{\pi }\right)\left(\frac{w}{4{\left(x-x_{c}\right)}^{2}+w^{2}}\right)$$

where *W*: FWHM (full width at half maximum), *y*_0_: offset in *y* (usually treated as zero), and *x*_c_: *x* which gives maximum *y* value.

(2) $$y=y_{0}+Ae-\left\{{\left(x-x_{c}\right)}^{2}/2w^{2}\right\}$$

where *W*: standard deviation (FWHM = 2.355*w*), *y*_0_: offset in *y* (usually treated as zero),and *x*_c_: *x* which gives maximum y value. Both functions gave comparable fittings as demonstrated in **Figure** **4** and listed in **Table** **1** for polyamide. Gaussian is known to be useful for the line shape analysis of broad peaks such as observed in wide‐band solid NMR. But as seen in Figure 4, Lorentzian can successfully fit the frequency profile. Thus, the prediction^[^ ^15^ ^]^ and confirmation^[^ ^16^ ^]^ of Lorentzian as a tool for analyzing the hyper‐CEST spectra is supported here for broad signals with more than 10 kHz FWHM. It is worth noting that broad lines may be a consequence of the factors such as chemical shift anisotropy (CSA)^[^ ^17^ ^]^ and exchange phenomena.^[^ ^18^ ^]^ CSA needs to be taken into account when the line shape is unsymmetric and very broad as observed in powder patterns. But such an unsymmetric profile was not evidenced in the present study. When the broadness comes from exchange phenomena of Xe between the sample and bulk gas, the line width of the sample will be increased at elevated temperatures. Since the line width observed at 90 °C is reduced by about 20% in polyamide (Table 1), the exchange phenomenon may not be related to the broadness in the present case.

**Table 1:** Analysis of the hyper‐CEST saturation frequency dependence of polymers.

Samplea)	Comment	Shape	xc	*w*	*A*	*R* ^2^ b)	χ^2^ b)	*δ* [ppm]	Diameterc) [nm]	FWHM [Hz]
Polyimide	22 °C No.1	Lorentz	2203 ± 158	10 536 ± 602	376 966 ± 15 457	0.954	1.53	206.9 ± 1.4	0.512 ± 0.003 (0.476)	10 536 ± 602
		Gauss	2291 ± 140	4590 ± 179	22 ± 1	0.958	1.37	207.7 ± 1.3	0.510 ± 0.003 (0.475)	10 810 ± 421
	22 °C No.2	Lorentz	2114 ± 176	10 805 ± 655	760 550 ± 32 969	0.944	7.29	206.0 ± 1.6	0.514 ± 0.004 (0.477)	10 805 ± 655
		Gauss	2106 ± 209	4882 ± 263	43 ± 2	0.917	10.8	205.9 ± 1.9	0.514 ± 0.004 (0.477)	11 497 ± 619
	22 °C average	Lorentz	2159 ± 45	10 671 ± 135	–	–	–	206.5 ± 1.1	0.513 ± 0.001 (0.476)	10 671 ± 135
	90 °C No.1	Lorentz	794 ± 78	8013 ± 258	906 051 ± 20 565	0.988	5.38	194.2 ± 0.7	0.543 ± 0.002 (0.492)	8013 ± 258
		Gauss	784 ± 168	3931 ± 186	67 ± 2	0.947	24.0	194.1 ± 1.5	0.544 ± 0.004 (0.492)	9258 ± 438
	90 °C No.2	Lorentz	817 ± 120	7910 ± 394	898 451 ± 31 558	0.972	13.0	194.4 ± 1.1	0.543 ± 0.003 (0.491)	7910 ± 394
		Gauss	746 ± 190	3890 ± 209	67 ± 3	0.933	31.4	193.8 ± 1.7	0.544 ± 0.005 (0.492)	9161 ± 493
	90 °C average	Lorentz	806 ± 12	7962 ± 52	–	–	–	194.3 ± 0.6	0.543 ± 0.001 (0.492)	7962 ± 52
Gelatin capsule	2024CE, No.1	Lorentz	717 ± 5	1470 ± 26	168 705 ± 2339	0.991	0.97	193.1 ± 0.1	0.546 ± 0.001 (0.493)	1470 ± 26
	2024CE, No.2	Lorentz	761 ± 13	1623 ± 69	115 281 ± 3874	0.958	1.79	193.7 ± 0.1	0.545 ± 0.001 (0.492)	1623 ± 69
	2017CE	Lorentz	1234 ± 29	1405 ± 118	103 098 ± 6972	0.923	8.36	197.8 ± 0.3	0.534 ± 0.001 (0.487)	1405 ± 118
TPU	22 °C	Lorentz	−86 ± 24	1122 ± 109	57 689 ± 4278	0.891	5.62	199.7 ± 0.2	0.529 ± 0.001	1122 ± 109
		Gauss	−81 ± 18	448 ± 27	33 ± 1	0.933	3.48	199.8 ± 0.2	0.529 ± 0.001	1046 ± 63
Silk fibroind)	22 °C	Lorentz	790 ± 15	1809 ± 73	58 657 ± 1987	0.975	0.25	192.9 ± 0.1	0.547 ± 0.001	1809 ± 73
		Gauss	789 ± 24	733 ± 40	20 ± 1	0.948	0.52	192.9 ± 0.2	0.547 ± 0.001	1712 ± 934
CNF‐A02d)	22 °C	Lorentz	−136 ± 47	2582 ± 238	134 570 ± 11 437	0.916	1.36	199.6 ± 0.4	0.529 ± 0.001	2582 ± 238
		Gauss	−137 ± 46	983 ± 80	33 ± 1	0.929	1.14	199.6 ± 0.4	0.529 ± 0.001	2295 ± 187

a) The saturation frequency dependence was measured with the saturation frequency increment 9.94 ppm (1100 Hz) for polyimide and 0.904 ppm (100 Hz) for others;

b) *R* ^2^ and χ^2^ denote coefficient of determination and residual sum of squares divided by degree of freedom, respectively;

c) Diameter for sphere. In parenthesis given the value for cylinder, for which errors are half of those of sphere;

d) Cited from ref. [11]. TPU: thermotropic polyurethane, CNF‐A02: cellulose nanofiber.

Duplicate measurements and fittings have established a very broad peak with the FWHM of about 96 ppm (11 kHz) and the chemical shift at 206.5 ± 1.1 ppm (Table 1). As it turned out, the signal has been incorporated into the baseline due to the very broad line widths of polyimide (Table 1).

In the present study, rf saturation was made by pulse mode. The saturation pulse used was gauss 6912 μs width which corresponds to 180° pulse with an excitation band width of 290 Hz.^[^ ^19^ ^]^ It is reported that the nominal band width becomes irrelevant when the exchange kinetics is in a relative fast regime and dominates the observed line width.^[^ ^20^ ^]^ Therefore, the repetitive many pulses with a narrow excitation bandwidth were efficient for establishing the very broad line that was hidden under the baseline as a result of a large line width amounting as much as 96 ppm (11 kHz) in case of polyimide.

When the measurement temperature was increased from 22 to 90 °C, the width was decreased to about 8 kHz, and the chemical shift moved toward high field by 12 ppm (Table 1). High field shift at elevated temperatures is common to ^129^Xe spins entrapped in polymers or other porous materials such as zeolites. A duplicate measurement revealed a high reproducibility of the chemical shift for the extraordinary broad peak: a broad peak with the FWMH amounting to 100 ppm was reproduced within the error of 1.1 ppm in the chemical shift at 22 °C. This demonstrates the value of the hyper‐CEST subtraction spectrum, which may be comprehended by taking into account the impact of peak phase adjustment on the top of peak, or chemical shift. Specifically, a slight variation in the phase of peaks, which form the envelope of a broad peak (see Figure 3), has no impact on the fitting outcome when the saturation frequency dependence is fitted by least squares method. In contrast, phase adjustment has a significant impact on the peak's top, which represents the chemical shift, when a very broad peak is directly observed. Such a case was realized in the standard hyper‐CEST experiments with CrA‐ma, cryptophane‐A‐monoacid, and CB6, cucurbit[6]uril, for which the line width was about 1.1 kHz.^[^ ^20^ ^]^ In the present study, this merit of the hyper‐CEST detection was established for the peaks with the line width amounting to as much as 96 ppm (11 kHz). The subtraction mode adopted in HCSS is useful for compensating for the fluctuations possibly occurring in the HP system in a relatively short period of time as well as the drift possibly occurring in the system through the whole experiment. This is quite helpful for detecting the top of very broad peak precisely and enhance the stability of HCSS. The stability is crucial for the determination of chemical shift of very broad signals for which changes in intensity are very small in the top region and liable to be affected by noise and/or drift, preventing a safe and sound determination of the point of highest intensity that corresponds to the chemical shift.

A particularly limited diffusion or short mean free path^[^ ^21^ ^]^ of the spin in polyimide is also supported by the chemical shift of 206.5 ppm, which is located in lower field than that seen with other well‐known polymers. The size of void space, or free volume, can be estimated according to the following equation,^[^ ^21^ ^]^

(3) $$\begin{array}{c} \delta s=\;243\left\{0.2054/\left(0.2054\;+\;\ell \right)\right\}\\ \ell =\left(D-0.44\right)/2,\text{ for sphere};\;\ell =D-0.44\text{ for cylinder } \end{array}$$

where *δs*, ℓ, and *D* are chemical shift of ^129^Xe, mean free path of ^129^Xe spins, and diameter of the voids space, respectively. The diameter estimated is 0.51 nm for sphere and 0.48 nm for cylinder space. Hitherto, the PALS has been reported for polyimide only in a limited case including the related polymers. This limited study is probably due to a special property of polyimide in which positronium formation is restrained preventing facile observation of the PALS signal.^[^ ^22^ ^]^ In the reported cases, the diameter of spherical free volume is 0.44 and 0.52 nm for a high‐barrier polyimide and Kapton, respectively,^[^ ^23^ ^]^ and 0.58–0.61 nm for three fluorinated polyimides.^[^ ^24^ ^]^ These values were obtained under the assumption of spherical cavity, and comparable to our result of 0.51 nm. Our values of 0.51 and 0.48 nm are very close to the van der Waals diameter of 0.44 nm for Xe atom, especially in the case of the cylinder space. Therefore, even though it is difficult to determine the precise geometry of the voids space at the nanoscale level, the remarkable broad peak with lower chemical shift found for the polyimide in the present study suggests cylindrical voids space in the polymer. Polyimides possess characteristic properties of high thermotolerance, high mechanical strength and high insulation property: it is used as flexible printed board for electronic devices, insulative protective films for semiconductor elements, and insulative protective films for artificial satellite or spacecraft. These characteristic properties may be said to come, at least partly, from the narrow void space as manifested in the present study.

**Figure 5:**
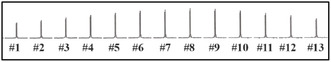
The saturation frequency dependence in HCSS of gelatin capsule. The saturation frequency was changed from 199.3 to 188.4 ppm in the increment of 0.904 ppm (100 Hz) from left (#1) to right (#13).

The gelatin samples measured in the present study was the gelatin capsule #2, Matsuya Co. Ltd., Japan, as listed in Japanese Pharmacopoeia. The hyper‐CEST saturation frequency dependence measured with the frequency increment of 9.94 ppm (1100 Hz) clearly showed a maximum intensity at the saturation frequency of 196.5 ppm (Figure S2, Supporting Information), although standard HP ^129^Xe spectra showed no signals assignable to ^129^Xe entrapped in gelatin. A more detailed measurement of the saturation frequency dependence (**Figure** **5**) was fitted by the least squares algorithm (Figure S3, Supporting Information). The line width was narrow similarly to the samples other than polyimide listed in Table 1.

An old gelatin capsule from 2017 CE that was kept on a shelf in our laboratory was also analyzed to see if the hyper‐CEST subtraction spectrum could be used to investigate the aging effects of gelatin. While there was no significant difference between the various lot numbers in 2024 CE, it was discovered that the gelatin capsules from 2017 CE displayed a low field shift of roughly 3–4 ppm in comparison to those from 2024 CE. This low field shift suggests cavity size reduction on aging (Table 1). The aging of gelatin is known to induce intermolecular cross links formed between amino acids and related to decreased gelatin dissolution.^[^ ^25^ ^]^ A MD (molecular dynamics) calculation supported more compact structure, i.e., tightness of protein structure as evaluated by the radius of gyration Rg, in the gelatin hydrogel in which covalent cross links are induced by transglutaminase.^[^ ^26^ ^]^ The low field shift observed in the present study is interpreted to indicate reduction in the cavity size or tightening of protein structure by the progressive cross links.

## 3 Conclusions

In this way, the proposed HCSS is demonstrated to disclose hidden or very weak signals of ^129^Xe embedded in various materials and to be a unique tool for evaluating properties and functions on the molecular level. This method can widen the applicability of ^129^Xe NMR to materials hitherto not accessible and would promote the academic as well as industrial research to develop new materials.

## 4 Experimental Section

4.1

4.1.1

##### Xenon Hyperpolarization and Delivery: Stopped‐*Flow Delivery from Continuously Recirculating Hyperpolarization System*

High‐content 70% Xe gas with 30% N_2_ was hyperpolarized in a continuous and recirculating mode through SEOP (spin‐exchange optical pumping) in a cell measuring 70 mm in diameter and 250 mm in length. The gas flow rate was about 100 mL/m, and ^129^Xe spin polarization was about 5–10% when the 25 W laser with a spectral width (FWHM) of less than 0.2 nm (Sultra‐50, QPC Lasers Inc., CA, USA) was used. The recirculating system was able to reduce the Xe gas consumption to as low as a few percent compared to the case without recirculation. The HP gas was directly flowed through a glass capillary into a 10 mm NMR tube wherein a polymer sample of about 0.1 g was included. Stopped‐flow was a mandatory scheme for observing the measurable stable hyper‐CEST signal.

**Figure 6:**
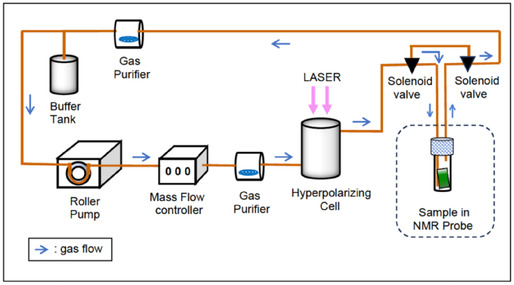
Schematic drawing of the recirculating gas flow in the hyperpolarization system.

In order to ensure a steady flow of gas through the hyperpolarizing cell and avoid any potential fluctuation in the spin polarization, the gas was passed through a by‐pass when stopped to a sample (**Figure** **6**).

After evacuating the whole system by a vacuum pump, high‐content 70% Xe gas with 30% N_2_ was added to a pressure near the atmospheric pressure and recirculated under flow control by the mass flow controller (Model 5100 Series, KOFLOC Co., Ltd., Tokyo) and roller pump (RP11, FURUE Co., Ltd., Tokyo). A buffer tank was attached to stabilize the flow of gas in the pipe line. Gas impurity contamination was prevented by passing the duplicate over the purifier (Na‐K alloy, Sigma‐Aldrich). The stopped‐flow was carried out by the two 3‐port solenoid valves whose ON‐OFF was controlled by the NMR pulse program through a spare switch attached to the NMR console. Almost all gas transmission pipelines were constructed of glass.

##### HCSS

Stopped‐flow hyper‐CEST pulse sequence sh2SatSub developed in previous research^[^ ^11^ ^]^ was applied in the following manner. See Figure 1 for the sequence. 1) HP gas is flowed to the sample in the predelay period *d1* and then stopped and changed to a bypass route. A waiting time *d4* is inserted after “Stop Flow.” 2) In the saturation period, *nprepul* pulses whose pulse width is *p1* are repeatedly applied with the delay time *d2*. In this case, the pulse pattern is gauss. 3) In the acquisition period, a shaped observation pulse whose pulse width is *pw* is applied followed by fid acquisition in the acquisition time *at*. In this case, the pulse pattern is sinc. 4) Subtraction is made in the following manner. First, fresh gas is delivered, and then the pulse sequence depicted in Figure 1 is applied by setting the saturation frequency at a reference frequency, and the fid signal is added to the computer memory (ct = 1). Next, fresh gas is delivered, and then the pulse sequence depicted in Figure 1 is applied by setting the saturation frequency at a measurement frequency. The resulting fid signal is subtracted from the computer memory (ct = 2). This add‐sub process is repeated by changing the measurement frequency stepwise over the total observation range to obtain the final saturation frequency dependence as depicted in Figure 2. Here, reference frequency is set at a mirror position of the measurement frequency with regard to the bulk gas signal, i.e., *f*_meas_–*f*_bulk _= *f*_bulk_–*f*_ref_.

It was not evidenced that the recirculating hyperpolarized gas affected the baseline in the following measurement. The magnetization is considered to be reduced to a negligibly small value when the gas returns to the hyperpolarizing cell through the pipeline, gas purifier, roller pump, and mass flow controller.

##### NMR Measurements

All NMR studies were performed using a 9.4 T Agilent Unity INOVA 400WB wide bore NMR spectrometer, Agilent Technologies, CA, USA. The ordinary HP ^129^Xe spectra were unable to detect any signals assignable to ^129^Xe entrapped in the polymer (Figure S1, Supporting Information).

The hyper‐CEST experimental conditions were as follows.

Figure 2, *sw* (spectral width): 10 kHz, at: 0.5 s, *pw*: 2048 μs sinc, *p1*: 6912 μs gauss, *d1*: 20 s, *d2*: 0.025 s, *d4*: 0.5 s, *tpwr*: 26 dB, *lb* (line broadening): 100 Hz, *ct* (number of completed transients): 2, *nprepul*: 10 k.

Figure 5, same as above except *nprepul*: 20 k.

##### Statistical Data Analysis

In the analysis of saturation frequency dependence in the hyper‐CEST experiments, a least‐squares fit based on the Levenberg–Marquardt algorithm on the program ORIGIN supplied from Lightstone Co., Tokyo, was used.

## Conflict of Interest

The authors declare no conflict of interest.

## Author Contributions

**Hideaki Fujiwara**: conceptualization, methodology, experiment and analysis, writing, funding acquisition. **Hirohiko Imai**: methodology. **Atsuomi Kimura**: methodology, experiment and analysis, funding acquisition. All authors have read and agreed to the published version of the manuscript.

## Supporting information

Supplementary Material

## Data Availability

The data that support the findings of this study are available from the corresponding author upon reasonable request.
