# Supplementary material for: Proposition of Hyper‐Chemical Exchange Saturation Transfer Subtraction Spectroscopy to Detect Very Weak and Broad Signals Hidden Under Baseline and Widen Range of Materials Accessed by Hyperpolarized 129Xe NMR
Source: Chemphyschem. 2025 Oct 26;26(23):e202500249. doi: 10.1002/cphc.202500249 (PMC12677713; doi:10.1002/cphc.202500249)
Supplement: Supplementary file 1 — Supplementary Material [file CPHC-26-e202500249-s001.pdf]

Supporting Information

Proposition of Hyper-CEST Subtraction Spectroscopy to Detect Very Weak and Broad Signals Hidden under Baseline and to Widen Range of Materials Accessed by Hyperpolarized ^{129}Xe NMR

Hideaki Fujiwara, Hirohiko Imai, Atsuomi Kimura**

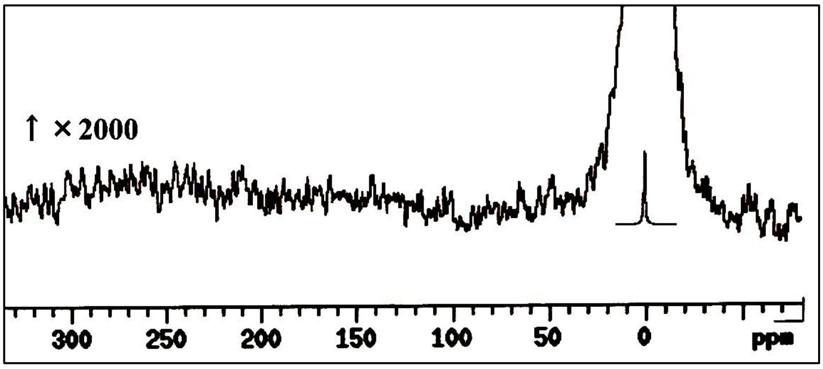

Fig. 1. Hyperpolarized ^{129}Xe NMR spectrum of polyimide tube measured in the standard mode.
sw:60 kHz, *at*:0.5 s, *pw*: 145 μs, *tpwr*: 35 dB, *d1*:2 s, *ct*: 8480, *lb*:100 Hz.

The original raw spectrum was measured with 60 kHz spectral width, *i.e.*, sw= 60 kHz, where the rf frequency offset was set so that the spectral center corresponds to 200 ppm from the bulk gas peak at 0 ppm. By using the long pulse, sensitivity in the central 200 ppm region is increased 40-fold compared to the peripheral 0 ppm region. Shown is the spectral part of interests ranging from about 300 to -80 ppm. The weak peak at 0 ppm is plotted under the amplitude reduced to 1/2000 of the strong peak.

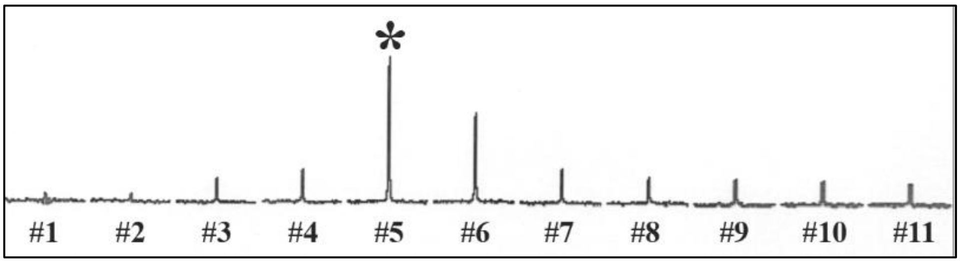

Fig. 2. The saturation frequency dependence in HCSS of gelatin capsule.

The saturation frequency was changed from 236.3 to 136.9 ppm in the increment of 9.94 ppm (1100 Hz) from left (#1) to right (#11). The * marked peak showed maximum intensity at 196.5 ppm, around which detailed measurement was performed in the increment of 0.904 ppm (100Hz) as shown in Figure 5 in the main text. Measurement parameters are same as in Figure 2 in the main text. Intensity is in arbitrary scale.

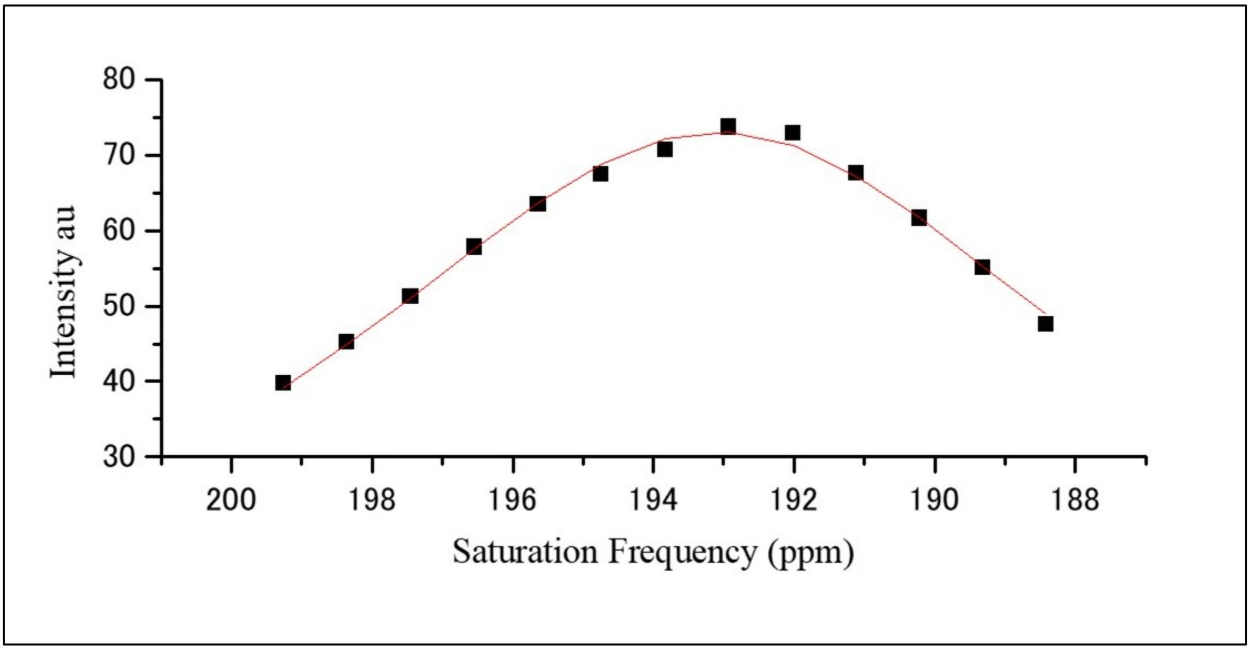

Fig. 3. Analysis of the saturation frequency dependence in HCSS of gelatin capsule. The curve denotes least squares fit by the Lorentz function.
